# Symptoms of depression and anxiety in Indonesian medical students: association with coping strategy and resilience

**DOI:** 10.1186/s12888-022-03745-1

**Published:** 2022-02-07

**Authors:** Adhitya Sigit Ramadianto, Irmia Kusumadewi, Feranindhya Agiananda, Natalia Widiasih Raharjanti

**Affiliations:** Department of Psychiatry, Faculty of Medicine Universitas Indonesia – Cipto Mangunkusumo Hospital, Jakarta, Indonesia

**Keywords:** Anxiety, Coping strategy, Depression, Undergraduate medical education, Medical students, Resilience

## Abstract

**Background:**

Depression and anxiety are prevalent mental health issues among medical students due to the various challenges during medical education. These issues affect not only their quality of life, but also their academic and professional development. Coping strategy and resilience are two factors that may influence students’ mental health outcomes. Data of medical student mental health in Indonesia is scarce, hampering efforts to systematically address the problem. Hence, this study aims to estimate the prevalence of depressive and anxiety symptoms in Indonesian medical students, and their association with coping strategy and resilience.

**Methods:**

Undergraduate medical students from each year of study (Preclinical Year 1 to 4, Clinical Year 1 and 2) in the Faculty of Medicine Universitas Indonesia were randomly selected to participate in this cross-sectional study. The study questionnaire included sociodemographic characteristics, Depression Anxiety Stress Scale (DASS) to measure symptoms of depression and anxiety, Brief COPE to measure coping strategy, and Connor-Davidson Resilience Scale (CD-RISC) to measure resilience. Scores of depression and anxiety symptoms were analyzed by comparing them between different sociodemographic groups and by measuring their correlation with coping strategies and resilience. Multiple regression analyses were conducted to identify predictors of depression and anxiety symptoms.

**Results:**

Among 532 respondents, 22.2% reported symptoms of depression and 48,1% reported anxiety, including 3.0% and 8.1% with extremely severe depression and anxiety, respectively. Students not living with immediate family had higher depression score; female students and those in Preclinical Year 1 and Clinical Year 1 showed higher anxiety scores. Dysfunctional coping strategies and lower resilience are predictors of higher depression and anxiety symptoms.

**Conclusions:**

Students show different levels of depressive and anxiety symptoms, signifying different levels of mental health support needs from universal mental health promotion to psychiatric treatment. Prevention programs can be targeted towards students with risk factors, such as not living with immediate family, undergoing first year of preclinical studies or clinical rotations, coping with dysfunctional strategies, and having low resilience. Additionally, medical educators must be aware of other, non-student factors that may impact student mental health, such as curriculum design and learning experience.

## Background

The medical education system is designed to equip students with the knowledge and skills to provide safe, high-quality medical care within the framework of a healthcare system [1]. In the process, students form their identity as physicians that conform to professional and legal standards [2]. Thus, the time spent in medical education can be an exciting period of personal and professional development.

Students’ physical and psychological well-being has been recognized as an important component in achieving those educational goals [3]. However, medical students face a myriad of challenges during their education that may pose a risk to their psychological well-being [4–6]. These challenges may arise from the medical education curriculum itself. Mastering all the required knowledge and skills in a relatively short period can lead to high academic workload, which may overwhelm students [4–6]. Additionally, parts of the workload may be deemed unnecessary or unrealistic [6]. The delivery of the curriculum, in the form of teaching and evaluation methods, can impact students’ psychological health as well [7, 8]. Students are also expected to adapt to different educational demands and settings, which become more pronounced when they undergo clinical rotations [5]. The hidden curriculum, composed of unwritten values, expectations, and power dynamics, can be another source of stress, especially when it exposes students to ethical conflicts [5]. Outright bullying or harassment against students remais prevalent too [5]. These stressors at school add to the stress from students’ personal life. On the other hand, medical students are often deprived of their social support network and opportunities to recuperate [4, 6].

Consequently, medical students often experience poor mental health, including symptoms of depression and anxiety. Two meta-analyses have found that, globally, more than 1 in 4 students report symptoms of depression, and up to 1 in 10 students report suicidal ideation [9, 10]. Another global meta-analysis found that 1 in 3 medical students experience symptoms of anxiety [11]. Longitudinal studies have revealed that students enter medical education with psychological well-being that is comparable or even better than the general population. However, their rates of depression and anxiety increase during their time in medical school [12, 13].

Mental health issues of medical students have gained significant attention, not only due to their prevalence, but also due to the short- and long-term consequences. Poor mental health may contribute to impaired academic performance and achievement of competencies [14]. It may also erode students’ empathy, ethics, and other humanistic qualities that are essential for medical practice [15, 16]. Furthermore, there are concerns that untreated mental health issues in medical students are the underlying cause of the poor mental health of physicians [17]. Ultimately, the mental health of current medical students will, through multiple pathways, shape the healthcare system of the future.

Coping strategy and resilience are two characteristics that are thought to moderate the influence of stressors on student mental health. Students may employ different coping strategies when dealing with different demands during their education. If the coping strategy is effective and suitable for the demands at hand and the available resources, it can confer protection. On the other hand, ineffective or maladaptive coping may be associated with negative mental health outcomes [18, 19]. Resilience is the ability to recover and grow from adversity through positive adaptation, without developing physical, psychological, or psychosocial disabilities [20, 21]. Resilient students can perceive problems as opportunities for growth, recognize available resources, organize themselves, and show creativity, optimism, and humour [20]. Furthermore, these two factors are of special interest for medical educators, as they are relatively amenable to psychological interventions to promote students’ mental health, compared to the rigid structure of curriculum and medical training [22, 23].

As reflected by the proliferation of research on this topic, the importance of medical students’ mental health has been widely recognized by medical educators. However, data from Indonesian setting is rather limited, which negatively affects how medical schools can respond to students’ mental health needs. While the Indonesian medical education system may share some characteristics with those of other countries, there are also considerable differences. For example, the education to be a general physician is taken at the undergraduate level, while specialist residencies are considered as optional post-graduate study. Indonesia also has its own set of expected physician competencies, as influenced national health policies and resources, and medical education culture that may affect the mental health of students. Reflecting Indonesian cultures in general, Indonesian medical education culture has been described as collectivist and hierarchical, with high power distance and emphasis on connectedness, which can influence how students experience their medical education. While this situation may be familiar in Asian countries, it is less so in other regions [24]. As a result, mental health programs that are successful elsewhere cannot be simply transplanted to Indonesian context.

To fill that information gap, this study aims to estimate the prevalence of depressive and anxiety symptoms among medical students in Indonesia. Coping strategy and resilience are also explored as possible factors that contribute to students’ mental health. Maladaptive coping strategies and lower resilience are hypothesized to be predictors of higher depressive and anxiety symptoms. The results of this study will give a picture of medical students’ mental health and their needs for support. Moreover, the findings of this study will add to the geographical diversity of studies on medical student mental health worldwide and provide an empirical foundation for programs to protect and improve the mental health of Indonesian medical students.

## Methods

### Context

This study was conducted in a public institution, the Faculty of Medicine Universitas Indonesia (FKUI), Jakarta. The medical doctor study program is undertaken at the undergraduate level for at least 11 semesters: 7 semesters of preclinical studies leading to Bachelor of Medical Science degree, and 4 semesters of clinical rotations which ends with the Medical Doctor degree. The preclinical phase is arranged as system-based modules. Students also get early exposure to clinical experience and field work. The clinical rotations are mainly conducted in Cipto Mangunkusumo Hospital (RSCM), the national referral hospital in Indonesia, along with other referral hospitals and primary healthcare centers around the city. In the first year of clinical phase, students undergo rotations in 4 10-weeks ‘major’ rotations in internal medicine, pediatrics, obstetrics and gynecology, and surgery. Afterwards, they rotate in 7 4-weeks ‘minor’ rotations. Students graduate after passing the national physician competency examination, composed of computer-based multiple-choice examination and objective structured clinical examination (OSCE).

FKUI also runs an international class program. The program curriculum is similar to the regular class, but English is used as language of instruction and there is an additional year between the end of preclinical studies and clinical rotations. During that year, students conduct research in partner universities in Melbourne, Australia or Newcastle, England to earn a degree from their respective institutions.

### Design

This cross-sectional study was conducted in the Faculty of Medicine Universitas Indonesia during the 2019–2020 academic year. The sample was selected using stratified random sampling, based on a complete list of students from the regular and international classes provided by faculty administration. For the regular class, 75 students were randomly selected from each year (Preclinical Years 1–4 and Clinical Years 1–2). Due to the smaller population of the international class, 80 students were randomized from preclinical phase (Preclinical Years 1–4) and another 80 students from the clinical phase (Clinical Years 1–2). Each selected student was given a written explanation about the nature and purpose of this study and were invited to participate. Those who respond to the invitation gave their written informed consent and filled the study questionnaire. Data collection had been completed before COVID-19 was declared a pandemic by the World Health Organization and the first case was found in the country.

The protocol of this study has been reviewed and given ethical clearance number ND-930/UN2.F1/ETIK/PPM.00.02/2019 by the Health Research Ethics Committee of FKUI-RSCM. All methods in this study were carried out in accordance to relevant guidelines and regulations.

### Measurements

The study questionnaire included questions on respondents’ sociodemographic characteristics and self-report instruments measuring symptoms of depression and anxiety, coping strategy, and resilience. The sociodemographic variables include age, gender, region of origin, accommodation during education, financing, type of high school, study program, and year of study.

Symptoms of depression and anxiety were measured by their respective scales in the Depression Anxiety Stress Scale (DASS). DASS had been adapted into Bahasa Indonesia with adequate validity and reliability [25]. The depression and anxiety scales each have 14 items, and respondents score them 0 to 3 according to their degree of agreement. The sum represents the severity of symptoms. DASS was designed to be analyzed numerically [26]. Nevertheless, previous studies have used categorization. For depression, scores 0 to 9 is normal, 10 to 13 mild, 14 to 20 moderate, 21 to 27 severe, and 28 or more is extremely severe. For depression, scores 0 to 7 is normal, 8 to 9 mild, 10 to 14 moderate, 15 to 19 severe, and 20 or more is extremely severe [12].

Coping strategy was measured using Brief COPE (Coping Orientation to Problems Experienced), which consists of 14 subscales for different coping strategies: active coping, planning, positive reframing, acceptance, humor, religion, using emotional support, using instrumental support, self-distraction, denial, venting, substance use, behavioral disengagement, and self-blame. The instrument had been adapted and validated for Indonesian population [27]. Each subscale consists of 2 items, scored between 0 to 3. Higher score on a subscale represents more frequent utilization of that coping strategy [28]. The 14 subscales can be grouped into problem-focused coping, emotion-focused coping, and dysfunctional coping [29]. Students were instructed to fill the Brief COPE according to their coping strategy when facing challenges or issues related to their medical education.

Last, resilience is measured using Connor-Davidson Resilience Scale (CD-RISC). The instrument is composed of 25 items, each scored between 0 to 4. Students with higher sum of all items are said to have higher resilience [30]. It had been translated into Bahasa Indonesia with adequate validity and reliability [31].

### Data analyses

Study data was managed using SPSS for Windows version 22. Descriptive statistics for categorical variables are reported as proportion. For numerical variables, the Kolmogorov–Smirnov test was used to assess distribution of data. Variables that follow normal distribution are reported as mean with standard deviation (SD) while variables that do not are reported as median with interquartile range (IQR). Comparison of depressive and anxious symptoms between two groups was carried out using unpaired t-test or Mann–Whitney test; whereas comparison between more than two groups was done by ANOVA or Kruskal–Wallis test. The relationship between symptoms of depression, symptoms of anxiety, coping strategy, and resilience was measured by correlation, reported as Pearson correlation coefficient or Spearman correlation coefficient. The level of statistical significance was set at *p* < 0.05. Subsequently, we performed multiple linear regression analyses to identify predictors of depression and anxiety symptoms by including variables with *p*-value < 0.2 in the bivariate analyses, and for correlations, a correlation coefficient of *r* > 0.2.

## Results

Out of the 610 randomly selected students, 532 responded (87,2% response rate) and filled the study questionnaire: 343 students from preclinical phase and 189 from clinical phase. The remaining selected students did not respond to the invitation to participate. The age of respondents ranges from 17 to 27 years old, with a mean of 20.65 (*SD* = 1,98). More than half (59.6%) of respondents is female. More than two-thirds come from the Greater Jakarta Area, and 44.2% do not live with their immediate family. Approximately 61.7% studied in public high school. Almost all respondents finance their studies independently without any financial aid.

Approximately 22.2% of students showed depressive symptoms, mostly in the mild and moderate category, with a median score of 4 (IQR 1 – 9). Meanwhile, symptoms of anxiety were reported by 48.1% of students. Similarly, most are in the mild and moderate range, with a median score of 7 (IQR 4 – 13) (Table 1). Among all students, 18,6% showed symptoms of both depression and anxiety. The scores for depressive and anxiety symptoms exhibit statistically significant correlation with moderate strength (*r* = *0.653; p* < *0.001*).

**Table 1:** Distribution of depressive and anxiety symptoms

	Depression		Anxiety	
	n	%	n	%
Normal	414	77.8	276	51.9
Mild	47	8.8	64	12.0
Moderate	46	8.6	98	18.4
Severe	9	1.7	51	9.6
Extremely severe	16	3.0	43	8.1

Students not living with their immediate family had higher score of depressive symptoms. Higher scores of anxiety symptoms were found in female students and students of different years, especially in the first year of preclinical studies and in the first year of clinical rotations (Table 2).

**Table 2:** Distribution of depressive and anxiety symptoms by sociodemographic variables

	*n*	*(%)*	Depression score *(median [IQR])*	*p value*	Anxiety score *(median [IQR])*	*p value*
All subjects	532	100	4 (1–9)	-	7 (4–13)	-
Gender						
Male	215	40.4	4 (1—8)	0.707	7 (4—11)	**0.027**
Female	317	59.6	4 (1—9)		8 (4—13)	
Region of origin						
Greater Jakarta	366	68.8	4 (1—9)	0.862	7 (4—12)	0.168
Java outside Greater Jakarta	96	18.0	3 (3—8)		7 (3—11)	
Other than Java	70	13.2	4 (1.75—9)		8 (4—15)	
Accommodation						
With immediate family	297	55.8	3 (1—8)	**0.041**	7 (4—12)	0.580
Not with immediate family	235	44.2	4 (2—9)		7 (4—13)	
Financing						
Out-of-pocket	479	90.0	4 (1—9)	0.716	7 (4—12)	0.799
Partial or full scholarship	53	10.0	3 (2 – 10.5)		7 (3—13,5)	
Secondary education						
Public high school	328	61.7	4 (1—8)	0.141	7 (4—12)	0.324
Private high school	159	29.9	5 (2—10)		8 (4—13)	
International high school	45	8.5	3 (1—7)		6 (4—10,5)	
Study program						
Regular class	376	70.7	4 (2 – 9.75)	0.099	7 (4—13)	0.220
International class	156	29.3	3 (1—8)		7 (4—11)	
Stage of study						
Preclinical	343	64.5	4 (1—9)	0.865	7 (4—13)	0.308
Clinical	189	35.5	4 (1—8)		7 (3—11)	
Year of study						
Preclinical Year 1	90	16.9	5 (2 – 9.25)	0.441	10 (6—16)	** < 0.001**
Preclinical Year 2	79	14.8	4 (2—9)		8 (4—13)	
Preclinical Year 3	92	17.3	3 (1 – 7.75)		6 (4—10)	
Preclinical Year 4	82	15.4	3 (2—9)		6 (4—11)	
Clinical Year 1	93	17.5	5 (1—10)		8 (3.5—14)	
Clinical Year 2	93	18.0	3 (1—7)		7 (3—10)	

Mann–Whitney U Test for comparison between 2 groups and Kruskal–Wallis Test for comparison between more than 2 groups; statistically significant results (*p* < 0.05) printed in bold

Students mainly utilize problem-focused and emotion-focused coping strategies, without any particular strategy being used more frequently. Most show statistically significant association with symptoms of depression, but the strength of correlation is low. Only ‘using emotional support’ has a relatively higher correlation coefficient (*r* = *-0.291; p* < *0.001*) (Table 3).

**Table 3:** Distribution of resilience and coping strategy, and their correlations with depressive and anxiety symptoms

	Median (IQR)	Depression		Anxiety	
		*r*	*p value*	*r*	*p value*
Resilience (0—100)	68 (58—77)	-0.428	** < 0.001**	-0.298	** < 0.001**
Coping strategy (0—6)					
*Problem-focused (0 – 18)*	*12 (11—14)*	*-0.188*	** < *0.001***	*-0.008*	*0.851*
Active coping	4 (4—5)	-0.160	** < 0.001**	-0.014	0.755
Planning	4 (4—5)	-0.094	**0.030**	-0.002	0.958
Instrumental support	4 (3—5)	-0.172	** < 0.001**	-0.009	0.840
*Emotion-focused (0 – 30)*	*20 (17—22)*	*-0.123*	***0.005***	*-0.017*	*0.692*
Acceptance	4 (4—5)	0.000	0.996	-0.019	0.655
Humor	3 (2—4)	0.177	** < 0.001**	0.054	0.211
Religion	4 (3—5)	-0.131	**0.002**	0.021	0.621
Emotional support	4 (3—5)	-0.291	** < 0.001**	-0.104	**0.017**
Positive reframing	4 (4—5)	-0.169	** < 0.001**	-0.090	**0.039**
*Dysfunctional (0 – 36)*	*14 (11—16)*	*0.461*	** < *0.001***	*0.378*	** < *0.001***
Self-distraction	4 (4—5)	0.115	**0.008**	0.084	**0.051**
Denial	1 (0—2)	0.268	** < 0.001**	0.230	** < 0.001**
Venting	3 (2—4)	-0.006	0.885	0.008	0.849
Substance use	0 (0—0)	0.228	** < 0.001**	0.201	** < 0.001**
Behavioral disengagement	1 (0—2)	0.414	** < 0.001**	0.311	** < 0.001**
Self-blame	3 (2—5)	0.438	** < 0.001**	0.378	** < 0.001**

Correlation shown using Spearman’s rank correlation coefficient; statistically significant results (*p* < 0.05) printed in bold

More variation is found in the dysfunctional coping strategies. Students use self-distraction, venting, and self-blame at a similar rate to problem-focused and emotion-focused coping. Less commonly employed coping strategies are denial, behavioral disengagement, and substance use. As a group, dysfunctional coping is significantly correlated with both symptoms of depression (*r* = *0.461; p* < *0.001*) and anxiety (*r* = *0.378; p* < *0.001*). For specific coping strategies, the strongest correlations with depressive and anxious symptoms are shown by self-blame and behavioral disengagement. Self-distraction has a significant but low-strength correlation, while venting is not correlated to either depressive symptoms or anxious symptoms (Table 3).

The median score of resilience, as measured by CD-RISC, is 68 (IQR 58 – 77). Higher resilience is moderately correlated with lower scores of depressive symptoms (*r* = *-0.428; p* < *0.001*) and anxious symptoms (*r* = *-0.298; p* < *0.001*) (Table 3).

We conducted multiple linear regression analysis for score of depressive symptoms with the following independent variables: accommodation, study program, secondary education, resilience, and the coping strategies emotional support, denial, substance use, behavioral disengagement, and self-blame. No significant collinearity was observed among the independent variables, with tolerance > 0.4 and variance inflation factor < 2. The model is statistically significant (*p* < 0.001; *F* = 47.626) and showed an adjusted *R*^*2*^ of 44,1%. The variables study program and secondary education did not reach statistical significance. Resilience and seeking emotional support are associated with lower scores of depression, while substance use, self-blame, denial, behavioral disengagement, and not living with family increase the score (Table 4).

**Table 4:** Multiple linear regression analysis for scores of depressive and anxiety symptoms

Variable	Unstandardized β (95% CI)	SE	Standardized β	*t*	*p* value
*Dependent variable: score of depressive symptoms*					
Constant	7.226 (3.352; 11.100)	1.972		3.665	** < 0.001**
Study program	-0.740 (-1.829; 0.349)	0.554	-0.048	-1.334	0.183
Secondary education	-0.027 (-0.794; 0.740)	0.390	-0.002	-0.069	0.945
Accommodation	1.410 (0.469; 2.324)	0.465	0.099	3.031	**0.003**
Resilience	-0.089 (-0.129; -0.049)	0.020	-0.174	-4.375	** < 0.001**
Coping: emotional support	-0.764 (-1.102; -0.426)	0.172	-0.152	-4.443	** < 0.001**
Coping: substance use	1.193 (0.744; 1.643)	0.229	0.175	5.215	** < 0.001**
Coping: denial	0.455 (0.024; 0.885)	0.219	0.072	2.073	**0.039**
Coping: behavioral disengagement	1.636 (1.161; 2.112)	0.242	0.278	6.765	** < 0.001**
Coping: self-blame	1.211 (0.888; 1.534)	0.164	0.256	7.365	** < 0.001**
*Dependent variable: score of anxiety symptoms*					
Constant	6.076 (2.304; 9.848)	1.920		3.165	**0.002**
Gender	1.627 (0.695; 2.559)	0.474	0.124	3.429	**0.001**
Region of origin	1.027 (0.384; 1.669)	0.327	0.114	3.138	**0.002**
Year of study	-0.490 (-0.755; -0.225)	0.135	-0.132	-3.626	** < 0.001**
Resilience	-0.081 (-0.121; -0.042)	0.020	-0.174	-4.104	** < 0.001**
Coping: substance use	1.395 (0.944; 1.846)	0.230	0.224	6.078	** < 0.001**
Coping: denial	0.769 (0.334; 1.204)	0.221	0.134	3.473	**0.001**
Coping: behavioral disengagement	0.723 (0.244; 1.202)	0.244	0.135	2.966	**0.003**
Coping: self-blame	1.035 (0.709; 1.360)	0.166	0.240	6.249	** < 0.001**

*CI* confidence interval; *SE* standard error; variables with *p* < 0.05 printed in bold

For symptoms of anxiety, the multiple linear regression analysis included the independent variables gender, region of origin, year of study, resilience, and coping strategies denial, substance use, behavioral disengagement, and self-blame. There was no significant collinearity among the independent variables. The resulting model was significant (*p* < 0.001; *F* = 31.755) and has an adjusted *R*^*2*^ of 31,7%. Along with female gender and originating from outside Jakarta, the coping strategies substance use, self-blame, denial, and behavioral disengagement predict higher anxiety; while resilience and increasing year of study are associated with lower anxiety scores. (Table 4).

## Discussion

The prevalence of depressive symptoms in this study at 22.2% is lower than the results of two global meta-analysis that each found the prevalence at 27.2% and 28% as well as the results of subgroup analysis from Asian countries (29.1% and 30.1%) [9, 10]. It is also lower compared to 3 studies using the same instrument and cut-off score (40.4%) [9]. Students not living with their immediate family have higher symptoms of depression. This finding may point to the loss of social support from family members that has been known to increase the risk of depression [32, 33]. Meanwhile, some other studies did not find similar association [34, 35]. Students may compensate the loss of support from immediate family through other sources of social support, such as peer groups and counsellors [32].

Symptoms of anxiety were found in 48.1% of students, much higher than the 33.8% prevalence from a global meta-analysis, and still higher compared to regions with the highest prevalence of anxiety: the Middle East (42.4%) and Asia (35.2%) [11]. The same meta-analysis also found a wide range of prevalence from studies using DASS, from 12.8% to 78.4% [11]. Female students showed higher scores of anxiety, which may reflect the difference of prevalence in the general population [36]. While the meta-analysis in medical students did not find the same association, a study from Brazil also found that female students have higher scores of state-anxiety and trait-anxiety [34]. Previous research has pointed at various possible causes for this sex difference in the prevalence of anxiety. Biological factors may include hormonal profile, neural circuitry, and neurotransmitter activity [37, 38]. These factors may also underlie psychological characteristics that predisposes to anxiety, such as neuroticism, ruminative tendencies, higher interpersonal sensitivity and attentional bias toward threats [37, 39, 40]. Women also face different life experiences and social expectations, which shape their brain and psychological development and, ultimately, their response to stressors [37, 40].

Higher scores of anxiety symptoms are found among students in the first year of preclinical studies and the first year of clinical rotations. Those years may represent periods of adaptation, respectively from secondary to tertiary education and from preclinical to clinical stage of medical education. They have very different learning activities and expectations, as well as educational environments with each of their written and unwritten rules. This pressure to adapt may become a considerable stressor for some students [5].

Respondents of this study show moderate level of resilience (68 [58–77]), slightly higher than the results of another study conducted in FKUI (64.76 to 67.60) [41] and also higher than a study from 3 medical schools in China (61.69 [SD = 10.55]) [42]. Higher resilience is moderately correlated with lower scores of both depression and anxiety symptoms. Existing studies have established similar correlations, albeit with different strengths of correlation [43–45]. More severe symptoms of depression and anxiety are more likely to be found among students with low resilience [45]. Resilience are also associated with better mental health indicators beyond depression and anxiety, such as quality of life, level of stress, burnout syndrome, and perception of educational environment. In many studies, resilience is conceptualized as a buffer or mediator between external stressors, student characteristics, and mental health outcomes [42–46].

Students employ problem-focused and emotion-focused coping strategies more frequently than dysfunctional coping. Among the dysfunctional coping strategies, only self-distraction is commonly used by students. This finding concurs with the results of another study from FKUI that only included first-year students [29]. Studies from other countries reveal similar trend of students relying on more adaptive or active coping methods such as acceptance, cognitive reappraisal, problem solving, planning, and seeking support [47, 48]. However, a study revealed that students’ use of distancing and self-control (keeping things to oneself) increases as they progress through their medical education [47].

Dysfunctional coping is clearly correlated with higher symptoms of depression and anxiety, with behavioral disengagement and self-blame showing the strongest association. Another study found that behavioral disengagement is the coping method that is more specifically used among students with mental health issues [48]. Students that employ ‘avoidant’ strategies (substance use, emotional distancing, self-harm) more frequently than or as frequently as ‘approach’ strategies (seeking help, seeking support, increasing effort) are more likely to have higher depression symptoms and to be burned out [32]. In contrast to previous studies, almost none of the respondents in this study resort to substance use as a coping strategy. This is a positive finding because besides being a sign of dysfunctional coping, substance use among medical students can be a risk factor of poor mental health and may compromise academic performance as well as patient safety [49, 50].

The results of this study showed that the respondents use various strategies in the problem-focused and emotion-focused coping groups with similar frequency. Individuals with a wide range of available coping strategies are said to possess a broad coping repertoire. It also hinted that students do not show a rigid preference for any particular coping strategy. In other words, they have a balanced coping profile [51]. A study in Singaporean medical students reveal that they are able to employ different coping strategies to manage different academic or relationship demands during the course of their medical education. They are also capable of judging whether certain coping strategies are effective or whether they should try alternative approaches [18]. Broad coping repertoire and balanced profile, coupled with the ability to accurately judge the effectiveness of a coping strategy, mean that students have the coping flexibility to match their strategy with the demands at hand [51]. Some studies suggest that coping flexibility is a stronger predictor of mental health compared to individual coping strategies [51, 52]. A study found that students in the clinical stage of medical education show more appreciation of the relationship between coping, stress, and mental health issues. Their understanding is also more grounded in real-life situations, instead of the more theoretical perspective of preclinical students [19].

In general, this study found that depression and anxiety are significantly correlated to resilience and coping strategy. However, the strength of correlations is in the low to moderate range, while the multiple linear regression analysis models can only explain less than half of the variance in outcome (44,1% and 31,7% for depression and anxiety scores, respectively). This finding can be explained by the fact that mental health depends on the complex interactions between countless internal and external factors, only some of which was explored in this study. In the lens of biopsychosocial model, students as individuals with their unique biological and psychological characteristics undergo the long process of medical education that exerts considerable pressure, which can be moderated by social support or exacerbated by the lack of social resources [53]. It is highly unlikely that any single factor can have a major impact. Instead, it is more likely that all the different factors influence mental health with smaller effects through different pathways. Along this vein, Dunn et al. offered the concept of a “coping reservoir” for medical student well-being. Student’s characteristics determine the internal structure of the reservoir, while negative and positive experiences can respectively deplete or replenish the reservoir. The interaction of those experiences and the reservoir itself shapes the outcome of student’s mental health [4].

The prevalence of depressive and anxiety symptoms in this study points to different levels of mental health support needs that should be anticipated by stakeholders. The majority of students have normal to low scores of depression and anxiety. However, it should be noted that low level or sub-threshold symptoms are significant risk factors for full-threshold mental disorder in the future. The risk is even more pronounced in late teenagers and young adults, the age range of respondents in this study [54, 55]. Additionally, those with subthreshold symptoms may already experience impairment in their role functioning and require professional help [55, 56]. Students without symptoms may also have risk factors for mental health issues, such as low resilience and a tendency for dysfunctional coping as found in this study. Therefore, it is important that medical schools regularly screen all students for mental health symptoms and risk factors. Results of screening can help schools formulate suitable mental health promotion programs and identify at-risk students who need more selective prevention programs [57]. For example, specific programs can be designed for students entering their very first year in medical school or those transitioning into clinical rotations.

Different types of mental health promotion and prevention programs can be found in the literature, as schools start appreciating the importance of mental health. A survey of medical schools in the United States found that more than half offer student well-being curriculum, with mandatory and optional activities. They may include peer support, stress management, counselling, physical activities, financial preparedness, recreational activities, and social events [58]. The design of wellness programs should center the lived experience and actual needs of students to ensure that the programs are effective and acceptable, and that resources are well-spent. Students and faculty may have different perspectives on well-being and ways to promote it [22]. Nevertheless, students are capable of identifying their needs and specify their preferred programs, or even generate their own ideas for mental health programs [59, 60].

Last, students who are experiencing significant symptoms of depression and anxiety should be provided access to effective and safe psychiatric treatment. An evaluation of mental health services for medical students in University of Cambridge found that such dedicated specialist service can effectively improve psychological well-being and functioning. Students also expressed their appreciation for the flexibility and accessibility of the service [59]. Barriers to access must be addressed, especially the stigma surrounding medical students and mental health issues [61].

The finding that mental health issues are correlated to resilience and coping strategy, both “internal” factors of the student, does not put the burden of mental health solely on students. Students are deeply affected by the social and educational systems they inhabit, over which they have little to no influence. Their “internal” factors, including resilience and coping, are also shaped by available resources and social environment. Thus, policy makers have a moral and ethical responsibility as well as the privilege to promote students’ mental health [62, 63].

Programs that are aimed to help students cope with the pressure of medical education can be beneficial. However, they exist in a paradigm in which that distress in medical education is a given and that medical schools may help students by “strengthening” them. Policy makers at all levels will eventually need to address the sources of distress in the education process itself if they wish to have a mental health program that is truly preventive [7]. A qualitative study that allowed students to generate their own ideas for well-being programs reported that students want changes to how their education is carried out [60]. Some suggested changes to the curriculum include pass/fail grading system, reducing contact hours, evaluating the depth of learning materials, and reformulating feedback given to students [7]. A systematic review found that, while better quality studies are needed, these interventions do promote students’ mental health [64].

This study is one of the few that have systematically assessed mental health issues among Indonesian medical students and possible risk factors. While studies from other countries may inform Indonesian medical education system, locally-produced studies are important to uncover the similarities and differences that can assist in adopting best practices into Indonesian context. Furthermore, the sample size of this study is relatively large and representative, as subjects are selected through probabilistic methods from each year of medical school.

On the other hand, the cross-sectional design of this study prevents us from making definitive conclusions about the direction of causality between symptoms and risk factors. For example, it is possible that depressive symptoms reinforce dysfunctional coping strategies, such as self-blame and behavioral disengagement, that are included among commonly used criteria for depressive disorders [65]. The timing of measurement may also contribute to the variation of symptom severity [66]. Lastly, this study may have limited generalizability to the whole medical student population in Indonesia as the sample was taken from only one medical school in Jakarta, out of more than 70 medical schools in the country. Further studies would need bigger, more representative samples to gain a better understanding of mental health issues among Indonesian medical students.

Future studies could utilize cohort design to analyze the fluctuations of depressive and anxiety symptoms, as well as dynamic risk factors, over time in medical school. That information would enable researchers to determine the presence of causality and its direction. Such studies could be integrated into the medical education system at each school. Baseline data can be collected at student intake, and then followed up with regular frequency until students graduate or even beyond graduation. They could also consider additional risk factors, including sociocultural factors. In light of the COVID-19 pandemic, this study provides data on Indonesian medical students’ mental health prior to the pandemic itself. While this study does not reflect current situation in the ongoing pandemic, it can serve as baseline data to evaluate its impact.

## Conclusions

Approximately 1 in 4 students report experiencing symptoms of depression, while almost 1 in 2 report symptoms of anxiety. Dysfunctional coping strategies, especially self-blame and behavioral disengagement, are predictors of depression and anxiety symptoms. Students with higher resilience experience fewer depressive and anxiety symptoms. Medical educators and policy makers need to anticipate the different levels of support that students may need, from mental health promotion, prevention of mental health issues, to specialist psychiatric treatment. Beyond interventions that target students, educators must also evaluate and make changes to the education system itself to reduce the risk of mental health issues.

## Data Availability

The datasets used and/or analysed in this study are available from the corresponding author on reasonable request.

## Abbreviations

FKUI: Faculty of Medicine Universitas Indonesia
RSCM: Cipto Mangunkusumo Hospital
DASS: Depression Anxiety Stress Scale
CD-RISC: Connor-Davidson Resilience Scale
IQR: Interquartile range
SD: Standard deviation
